# Morpho-orthographic segmentation on visual word recognition in Brazilian Portuguese speakers

**DOI:** 10.1186/s41155-024-00331-0

**Published:** 2024-11-04

**Authors:** Humberto dos Reis Pereira, Francis Ricardo dos Reis Justi

**Affiliations:** 1 Graduate Program in Psychology, Cognition and Language Research Group, Federal University of Juiz de Fora, Juiz de Fora, Brazil; 2 Department of Psychology, Cognition and Language Research Group, Federal University of Juiz de Fora, Juiz de Fora, Brazil

**Keywords:** Morphological processing, Visual word recognition, Morphological decomposition, Reading, Morphology

## Abstract

**Objective:**

Studies have demonstrated a morphological decomposition process in the initial stages of visual word recognition based on orthographically defined morphemes and independent of semantic and lexical information. The present study sought to investigate this phenomenon in adult Brazilian Portuguese-speaking readers.

**Method:**

Participants performed a lexical decision task preceded by primes at two Stimulus Onset Asynchrony (SOA): 33 or 250 ms. The primes had one of the following relations with the target word: morphological (porteiro [DOORKEEPER]–PORTA [DOOR]), morpho-orthographic (cordeiro [LAMB]–CORDA [ROPE]), orthographic (abril [APRIL]–ABRIU [OPENED]), semantic (abelha [BEE]–MEL [HONEY]), or no relation (pessoa [PERSON]–DADO [DICE]).

**Results:**

Priming effects were observed for the morphological and semantic conditions at both SOAs but not for the orthographic and morpho-orthographic conditions.

**Conclusion:**

Our results suggest that semantic representations mediate morphological priming in Brazilian Portuguese since the early stages of visual word recognition.

## Introduction

*Morphological processing* is usually defined as the implicit use of morphological structures during reading (Law et al., 2018). Over the past few decades, there has been a concerted effort to understand its role in visual word recognition (for a review, see Amenta & Crepaldi, 2012). In particular, studies investigating the processing of morphologically complex words have indicated the presence of a morphological decomposition process in the early stages of visual recognition based on the orthographic structure of the morphemes (Amenta et al., 2020; Beyersmann et al., 2016; Grainger & Beyersmann, 2020; Heyer & Kornishova, 2018; Rastle & Davis, 2008; Tseng et al. 2020). However, to our knowledge, no studies in the literature have investigated this phenomenon with speakers of Brazilian Portuguese. Thus, the present study aimed to investigate if there are morphological and morpho-orthographic segmentation effects on visual word recognition in Brazilian Portuguese adult readers. Below, we review the seminal studies on the topic and present the rationale for the present study.

Studies employing lexical decision tasks reported that the brief presentation of a morphologically related word (prime) enhances the speed and accuracy of the following target word (Amenta & Crepaldi, 2012; Mousikou & Schroeder, 2019). However, studies with adult participants have shown that facilitation occurs not only for pairs of words with a transparent morphological relationship (e.g., darkness-dark) but also for pairs of words with only a resemblance of a morphological relationship (e.g., corner-corn), referred to as morpho-orthographic pairs (for a review see Rastle & Davis, 2008).

For instance, Rastle et al. (2004) asked participants to perform a lexical decision task with primes lasting 42 ms, organized in three conditions according to its relation with the target word: (1) morphologically transparent (e.g., cleaner-clean); (2) morpho-orthographic (e. g. corner-corn) and; (3) no semantic or morphological relationship (e.g., brothel-broth). Priming effects were observed for the first two conditions, compared with the third. These results have found support in different studies that have shown no differences between morphologically transparent and morpho-orthographic conditions (Beyersmann et al., 2015, 2016; Grainger & Beyersmann, 2020; Heyer & Kornishova, 2018; Kazanina, 2011; Lázaro et al., 2021; McCormick et al., 2008; Smolka et al., 2019; Tseng et al., 2020), suggesting the presence of a morphological segmentation process in the early stages of visual word recognition in adults based in morpho-orthographic structures. This perspective continues to be influential in discussions about how morphological processing occurs in different languages and age groups (Cayado et al., 2023; Ciaccio et al., 2020; Creemers et al., 2020; Fleischhauer et al., 2021).

An essential factor in priming studies is the Stimulus Onset Asynchrony (SOA), the interval between the prime and the target word’s presentation. Studies have shown that facilitation effects for words that share a morphological relationship with their primes are observed in both short and long SOAs (above 200 ms). In contrast, for the morpho-orthographic condition, facilitation effects appear only in short SOAs, while for the semantic condition, facilitation effects appear only in long SOAs when usually the prime is consciously perceived (Heyer & Kornishova, 2018; Quémart et al., 2011; Rastle et al., 2000).

The above results suggest a blind morpho-orthographic segmentation process operating in the early stages of visual word recognition, breaking words into parts considering only their orthographic similarity with morphemes and being insensitive to the semantic information. In this way, words would be decomposed into their morphemic units regardless of access to their semantic representation, which would explain the absence of differences between morpho-orthographic and morphologically transparent conditions in studies that used priming. Nevertheless, the results found by Crepaldi et al., (2010a), Diependaele et al. (2009) and Feldman et al. (2009) raise questions about the extension of morpho-orthographic processes. In the study by Crepaldi et al., (2010a), facilitation effects were observed in the priming task only when the prime was the irregular inflected form of the target verb (e.g., fell–fall), a condition in which both prime and target shared the meaning. However, no facilitation was observed when the prime was only orthographic similar (e.g., fill–fall) or when the words were unrelated but compatible with an orthographic pattern (e.g., book–bake). Additionally, the study byDiependaele et al. (2009) carried out with Dutch speakers observed that when primes were presented for 40 ms, only the condition of morphologically related words showed priming effects, these not being observed for the morpho-orthographic or orthographic conditions.

Finally, Feldman et al. (2009) challenged the decompositional view proposed by Rastle and Davis (2008) by indicating a greater magnitude of priming effects for the morphologically transparent condition compared with the morpho-orthographic condition in 16 studies published up to that moment, as well as in the results of the experiment carried out by the authors themselves. However, Davis and Rastle (2010) indicated that the results found by Feldman et al. were due to methodological differences, such as nonsystematic orthographic alterations between the prime and target, and ambiguous items presenting different affixes that could be decomposed into various bases in the morpho-orthographic condition. Despite that, in the following years, more studies started to support Feldman et al. by indicating that semantic information plays a role in morphological processing (Amenta et al., 2015; Beyersmann & Grainger, 2018; Feldman et al., 2015; Jared et al., 2017; Schmidtke et al., 2017).

### Linguistic variations in morphological processing

The findings above suggest that adult readers carry out a morphological decomposition process in the earliest stages of visual recognition. However, there are questions about the proposal, such as whether this segmentation process occurs based only on morpho-orthographic structures and is blind to semantic information. We should point out that the studies are mainly carried out with native English speakers, a language with low grapheme-phoneme transparency (Borgwaldt et al., 2005; Borleffs et al., 2019) and considered morphologically poor in comparison to French, for example (morphologically complex words in French: 75%, English: 55%; Casalis et al., 2015).

The study by Beyersmann et al. (2020), which was carried out with German and French speakers, suggested differences in the extent of the use of morphology during reading depending on the language. The authors asked participants to respond to a lexical decision task with pseudowords formed by real and non-real roots and suffixes. Although participants from both languages showed longer reaction times and error rates when pseudowords composed of a stem were presented, the magnitude of these effects was greater in German-speaking adults, a language with greater morphological productivity than French. In the review by Rastle and Davis (2008), although most studies focused on English speakers, variations in the magnitude of priming effects for words with apparent morphological relationships but lacking semantic relationships were observed across English, Russian, Dutch, and French. These observations indicate potential disparities in morphological processing based on the specific language under investigation.

In the case of Brazilian Portuguese, the language can be classified as intermediate on the spectrum of grapheme-phoneme transparency, positioned between languages with very inconsistent orthographies, such as English, and languages with more transparent orthographies, such as Italian (Borgwaldt et al., 2005; Lima & Castro, 2010; Seymour et al., 2003). One way to classify the transparency level of a language is based on the rate of simple correspondences, where a letter is mapped to a single sound, versus complex correspondences, where different letters are mapped to the same phoneme (Schmalz et al., 2015). Brazilian Portuguese has a considerable rate of simple correspondences with a few contextual rules regulating the pronunciation of some letters. For example, the letter < s > between vowels is mapped to the phoneme /z/, making it relatively transparent (Justi et al., 2023). In this context, Brazilian Portuguese readers might rely less on larger units, such as morphemes, and more on letter-sound decoding during reading since grapheme-phoneme patterns are sufficiently regular to enable effective reading.

### Morpho-orthographic processing in Portuguese

In Portuguese, to our knowledge, few studies have investigated the phenomenon of morphological decomposition. The study by Pinto (2017), carried out with European Portuguese speakers, focused on the effect of suffixes on morphological decomposition processes. The study presented primes for 50 ms and 150 ms, comparing pairs of words with the following relations: morphological (e.g., mineiro [MINER]–carteiro [MAILMAN]), morpho-orthographic (e.g., mineiro [MINER]–solteiro [SINGLE]), and no relation (e.g., mineiro [MINER]–caneta [PEN]). However, contrary to expectations, compared to the unrelated condition, there were no priming effects for either condition (morphological or morpho-orthographic) in both priming durations. 

Regarding Brazilian Portuguese, Garcia et al. (2012) conducted a lexical decision task with priming, in which participants were presented pairs of words with a morphological relationship (e.g., fila [QUEUE]–fileira [ROW]), a semantic relationship (e.g., ordem [ORDER]–fileira [ROW]), a phonological relationship (e.g., filé [FILLET]–fileira [ROW]), and unrelated pairs (e.g., mato [GRASS]–fileira [ROW]). Primes were displayed for 38 ms. Significant priming effects were observed only for the morphological condition in comparison with the unrelated condition, with the items of the morphological condition showing shorter reaction times. This result suggests that a morphological decomposition process may occur in the early stages of visual word recognition in Brazilian Portuguese. However, since the authors did not include a morpho-orthographic condition, it is not clear whether this effect is due to morpho-orthographic aspects, as suggested by Rastle et al. (2004), for example, or due to morpho-semantic aspects. Nevertheless, some considerations must be made regarding the study by Garcia et al. (2012). The number of items presented to the participants was considerably low (four items per condition), which can influence statistical power and the experiment’s capacity to detect significant statistical differences among conditions. In addition, the authors did not report having controlled the number of orthographic neighbors, a variable that influences visual word recognition in many languages, including Brazilian Portuguese (Andrews, 1997; Fiebach et al., 2007; Grainger et al., 2005;Justi and Roazzi, 2012; Xiong et al., 2021). Therefore, the number of orthographic neighbors may be a confounding variable, making it difficult to interpret the data.

## Current study

Studying how morphological decomposition occurs in adults can help understand the mental lexicon’s organization in proficient readers and the impact of linguistic variables. This investigation enables the establishment of explanatory models for morphological segmentation processes. These models, in turn, can contribute to understanding the developmental stages of morphological processing from early literacy to adult proficiency. Moreover, comprehending how morphological processing manifests in proficient adult readers aids in identifying whether each language possesses unique mechanisms for processing complex words or if a general learning mechanism exhibits language-specific variations. 

Considering the above, the present study aimed to investigate if there are morphological and morpho-orthographic segmentation effects on visual word recognition in Brazilian Portuguese adult readers. Specifically, we sought to analyze differences in priming effects among pairs of words that shared morphological or morpho-orthographic or semantic relationships compared to those in unrelated and orthographic conditions. The orthographic condition ensures that any observed effects in the morphological or morpho-orthographic conditions are not only due to orthographic similarity between the prime and the target.

Our initial hypotheses were that facilitatory priming effects (shorter reaction times and greater accuracy) would be observed when primes were displayed for short SOAs and had morphological or morpho-orthographic relationships compared to an unrelated condition and an orthographic condition. Similarly, we expected facilitatory effects when primes were displayed for long SOAs and had morphological or semantic relationships compared to an unrelated condition and an orthographic condition.

## Method

### Participants

Eighty-one undergraduate Psychology students (54 women, mean age = 20.4; SD = 2.68) enrolled in a Brazilian federal university participated in the experiment. All participants had Brazilian Portuguese as their native language and signed an Informed Consent Form. Participants were randomly assigned to one of two SOA conditions: short–33 ms (41 participants, mean age = 19.8, SD = 1.89) and long–250 ms (40 participants, mean age = 20.9, SD = 3.22). This study was approved by the Ethics Research Committee of the participants' university.

### Materials

The experimental stimuli comprised 100 pairs of words selected from the “*Léxico do Português Brasileiro”* (Estivalet & Meunier, 2015), corresponding to five experimental conditions (20 pairs per condition): morphological, morpho-orthographic, orthographic, semantic, and unrelated. In the morphological condition, pairs of words that shared a morphological transparent relationship (e.g., porteiro [DOORKEEP]–porta [DOOR]) were selected. While in the morpho-orthographic condition, pairs that do not share a semantic relationship but appear to have a morphological structure (e.g., cordeiro [LAMB]–corda [ROPE]) were selected. In the orthographic condition, pairs of words that shared an orthographic but not semantic or morphological relationship (e.g., girafa [GIRAFFE]–gira [SPIN]) were selected. Only pairs of words that shared a semantic but not orthographic or morphological relationship (e.g., abelha [BEE]–mel [HONEY]) were selected for the semantic condition. Finally, pairs of words that do not share orthographic, morphological, or semantic relationships were selected for the unrelated condition (e.g., medalha [MEDAL]–agenda [SCHEDULE]). Semantic similarity shared by each pair of words was assessed through the LX-SemanticSimilarity (https://portulanclarin.net/workbench/lx-semsim/) (Rodrigues et al., 2016). Characteristics of the stimuli can be found in Table 1.

**Table 1:** Mean, standard deviation, kurtosis, and skewness for the word pairs

Variable	Unrelated	Morphological	Morpho-orthographic	Orthographic	Semantic
Target					
Frequency	4.45 (0.42); − 0.09/ − 0.05	4.49 (0.45); − 1.68/3.35	4.48 (0.37); − 0.32/ − 0.92	4.47 (0.37); − 0.68/ − 0.24	4.52 (0.35); − 0.47/ − 0.15
Length	4.9 (0.85); − 0.36/ − 0.30	5 (0.79); 1.39/2.98	5.05 (0.99); 0.95/3.7	5.2 (0.77); 0.40/0.36	4.7 (1.17); − 0.43/ − 1.28
Orthographic neighbors	12.3 (8.28); 0.49/ − 1.28	11.9 (8.45); 0.92/ − 0.23	12.8 (6.05); 0.20/ − 0.76	11.3 (8.64); 1.23/0.66	11.8 (12.5); 1.65/2.17
Prime					
Frequency	4.26 (0.71); − 0.70/ − 0.42	3.30 (0.62); − 0.50/ − 0.50	3.94 (0.70); − 0.50/1.33	3.72 (1.14); 0.09/ − 1.23	4.10 (0.69); − 0.50/0.22
Length	6.95 (0.83); 0.1/ − 1.52	8.15 (0.74); 1.44/2.89	7.15 (1.27); 1.24/3.53	6.90 (1.07); − 0.35/ − 1.25	6.80 (1.40); 0.14/ − 1.08
Orthographic neighbors	3.65 (2.78); 0.29/ − 0.12	2 (2.08); 1.13/0.64	2.50 (1.54); 0/ − 1.61	3.70 (4.14); 1.93/4.67	3.25 (2.97); 0.96/ − 0.46
Semantic similarity prime-target	0.17 (0.06); 0.97/0.1	0.4 (0.12); − 0.11/ − 0.93	0.2 (0.07); − 0.65/0.27	0.2 (0.05); − 0.02/1.01	0.47 (0.07); 0.37/ − 0.13
Orthographic Overlap prime-target	0.4 (0.60); 1.24/0.78	4.2 (0.83); 0.8/0.72	4.5 (0.94); 0.67/1.28	4.15 (0.67); − 0.17/ − 0.55	0.5 (0.76); 1.99/5.13

Standard deviations were presented in parentheses, followed by skewness and kurtosis values. Orthographic frequency in Zipf scale (van Heuven et al., 2014), length in letters, and orthographic neighbors were taken from the “*Léxico do Português Brasileiro*” (Estivalet & Meunier, 2015). Semantic similarity between prime and target was calculated through LX-SemanticSimilarity (Rodrigues et al., 2016)

Target words had an average frequency of 4 on the Zipf scale, making them relatively frequent. They averaged around five letters in length and had approximately 12 orthographic neighbors. Since none of the experimental stimuli showed normal distribution by the Shapiro–Wilk test (all *p*s < 0.05), non-parametric Kruskall-Wallis tests were performed to test differences among the experimental conditions. Posthoc DSCF (Dwass-Steel-Critchlow-Flinger) for univariate comparisons were performed when necessary. As described in Table 1, no statistical differences were found among the targets regarding frequency, *χ*^2^(4) = 0.866, *p* = 0.929, length, *χ*^2^(4) = 1.856, *p* = 0.762, and number of orthographic neighbors, *χ*^2^(4) = 2.727, *p* = 0.605. In Brazilian Portuguese, morphologically complex words tend to be longer and less frequent than morphologically simple words that were used in the other experimental conditions. Despite an exhaustive search in “*Léxico do Português Brasileiro*” (Estivalet & Meunier, 2015), we could not identify primes for the morphological condition that were equivalent in length and frequency to the primes of the other experimental conditions. As a result, the primes in the morphological condition were less frequent than those in the unrelated and semantic conditions (*p*s < 0.007) and were longer compared to the other conditions (*p*s < 0.01). No differences were observed for the numbers of orthographic neighbors (*p* = 0.39). As for the semantic similarity, as expected, differences between the experimental conditions were also observed, *χ*^2^(4) = 64.373, *p* < 0.001: morphological and semantic conditions presented higher semantic similarity between the prime and the target than the other conditions (*p*s < 0.001) but did not differ from each other (*p* = 0.99). As we expected, differences were observed for orthographic overlap between prime and target, *χ*^2^(4) = 75.629, *p* < 0.001, with morphological, morpho-orthographic, and orthographic conditions showing greater overlap compared to unrelated and semantic conditions (*p*s < 0.001), but not differing from each other (*p*s > 0.64).

One hundred pseudowords were created for the lexical decision by changing one letter of the target words. This is a standard procedure commonly used in studies evaluating morphological processing (e.g., Beyersmann et al., 2021; Diependaele et al., 2009; Lázaro et al., 2016; Longtin et al., 2003). The primary reason for employing this procedure is to create a task that minimizes the use of strategies by participants. Explanatory models of the lexical decision task (e.g., Dufau et al., 2012; Norris, 2006; Ratcliff et al., 2004) suggest that the less similar a stimulus is to a real word, the easier it is for participants to respond that the stimulus presented was not a word. Therefore, using pseudowords created by changing one letter in a real word increases the difficulty, preventing participants from responding based solely on the stimulus’ global similarity to a real word. 

Orthographic neighbors for the pseudowords were obtained from N-Watch (Davis, 2005). No differences between targets and pseudowords were observed for the number of orthographic neighbors, *U*(198) = 4454, *p* = 0.18, and length, *U*(198) = 5000, *p* = 1. Each pseudoword was preceded by a prime equivalent of the experimental primes in length, *U*(198) = 4612, *p* = 0.33, orthographic neighbors, *U*(198) = 4707, *p* = 0.47, and frequency, *U*(198) = 4855, *p* = 0.71. The pseudowords primes had the same proportion of morphologically complex words as the experimental conditions.

### Procedures

The procedures adopted were adapted from the seminal study of Rastle et al. (2000). Lexical Decision Tasks were carried out on five Dell® Inspiron 3647 computers (800 × 600 resolution with a 60-Hz refresh rate). The E-Prime® 2.0 software was used for stimuli presentation, reaction times, and accuracy acquisition. Participants sat approximately 40 cm from the monitor, which could vary according to their posture. Primes were displayed for 33 or 250 ms in uppercase (the durations were defined according to the refresh rate of the monitor used), font Courier New 18, preceded by a mask “########” of 500 ms duration and followed by the target in lowercase, which remained on the screen until the participant gave an answer or 3 seconds had passed. The prime-target pairs were randomly presented. The participants were instructed to answer as quickly and accurately as possible whether or not a stimulus displayed was a real word, using a keyboard with a key marked in red for negative answers and another marked in green for positive answers. Unlike the 33 ms condition, an exposure time of 250 ms is sufficient for proficient readers to identify the presented prime word. Therefore, following Rastle et al. (2000), to prevent participants from responding to the primes, those assigned to the 250 ms SOA condition were informed that, before each letter sequence, a word that could or could not help in the task would be briefly displayed. No information was given about the prime for participants assigned to the 33 ms SOA condition. Participants received twelve practice trials before the actual experimental task. The experimental task had two independent variables (prime type: morphological, morpho-orthographic, orthographic, semantic, and unrelated; and SOA: 33 and 250 ms) and two dependent variables (reaction time and accuracy).

### Data analysis

For reaction time analysis, wrong answers were excluded (5.84%), and a semi-restricted correction was adopted (Perea, 1999): reaction times two standard deviations under or above the mean of each participant were replaced by the respective cutoff value (mean ± 2 standard deviation). This procedure changed 3.48% of the data. Reaction times were logarithmically transformed to correct the skewness and analyzed through a mixed repeated measure analysis of variance (ANOVA), with one between-subjects factor (SOA) and one within-subjects factor (prime type). Normality was assessed by the Shapiro–Wilk test, which shows the normality of the reaction time distribution of all conditions (all *ps* > 0.05). No sphericity problem was observed using the Mauchly test (*p* = 0.086). In contrast, the Levene test identified heterogeneity of variances only for the reaction time of the morphological condition (*p* = 0.02). In all other conditions, the assumption of homogeneity was not violated (*p* > 0.05).

## Results

The lexical decision task’s reaction times and error rates can be found in Table 2. Statistical significant differences were found for prime type, *F*(4, 316) = 63.17, *p* < 0.001, *η*^*2*^*p* = 0.444, and the interaction between prime type and SOA, *F*(4, 316) = 4.41, *p* = 0.002, *η*^*2*^*p* = 0.053. No main effect of SOA was observed, *F*(1, 79) = 3.07, *p* = 0.084, *η*^*2*^*p* = 0.037. For the variables prime length and prime frequency, which were not possible to control during stimuli selection, Pearson correlation analyses were performed. For short SOA, there were no significant correlations between both variables and reaction times (*p*s > 0.11). However, for long SOA, a significant correlation was observed between reaction time and prime frequency, *r* = 0.240, *p* = 0.016, indicating that words preceded by frequent primes were answered slower. No significant correlation was observed for prime length (*p* > 0.09).

**Table 2:** Reaction times (milliseconds) and error rates (percentage) by prime type and SOA

	Unrelated	Morpho-orthographic	Morphological	Orthographic	Semantic
SOA 33 ms					
RT	715 (107)	747 (119)	681 (94)	738 (109)	681 (102)
%Error	5.5 (6.9)	9 (7.3)	3.4 (5.1)	9.8 (8.5)	3.3 (4.7)
SOA 250 ms					
RT	780 (152)	789 (162)	710 (146)	819 (164)	738 (149)
%Error	5.1 (5.6)	7.9 (5.9)	2.1 (4.1)	10.6 (8.2)	1.6 (3.3)

Standard deviations were presented in parentheses

*RT *Reaction time

Given the presented hypotheses, planned comparisons were conducted to compare the experimental conditions. When the SOA was 33 ms, items of the morphological condition were identified faster than items of the unrelated condition, Δ = 34 ms, *t*(40) = 4.04, *p* < 0.001, *d* = 0.631. A priming effect was also observed for the semantic condition, Δ = 34 ms, *t*(40) = 5.96, *p* < 0.001, *d* = 0.932, in comparison with the unrelated condition. No differences were observed between morphological and semantic conditions, Δ = 0.54 ms, *t*(40) = 0.0806, *p* = 0.936, *d* = 0.012. Inhibitory effects in comparison with the unrelated condition were identified for the morpho-orthographic, Δ = 32 ms, *t*(40) = 4.3966, *p* < 0.001, *d* = 0.686, and orthographic conditions, Δ = 23 ms, *t*(40) = 2.90, *p* = 0.006, *d* = 0.454.

**Fig. 1:**
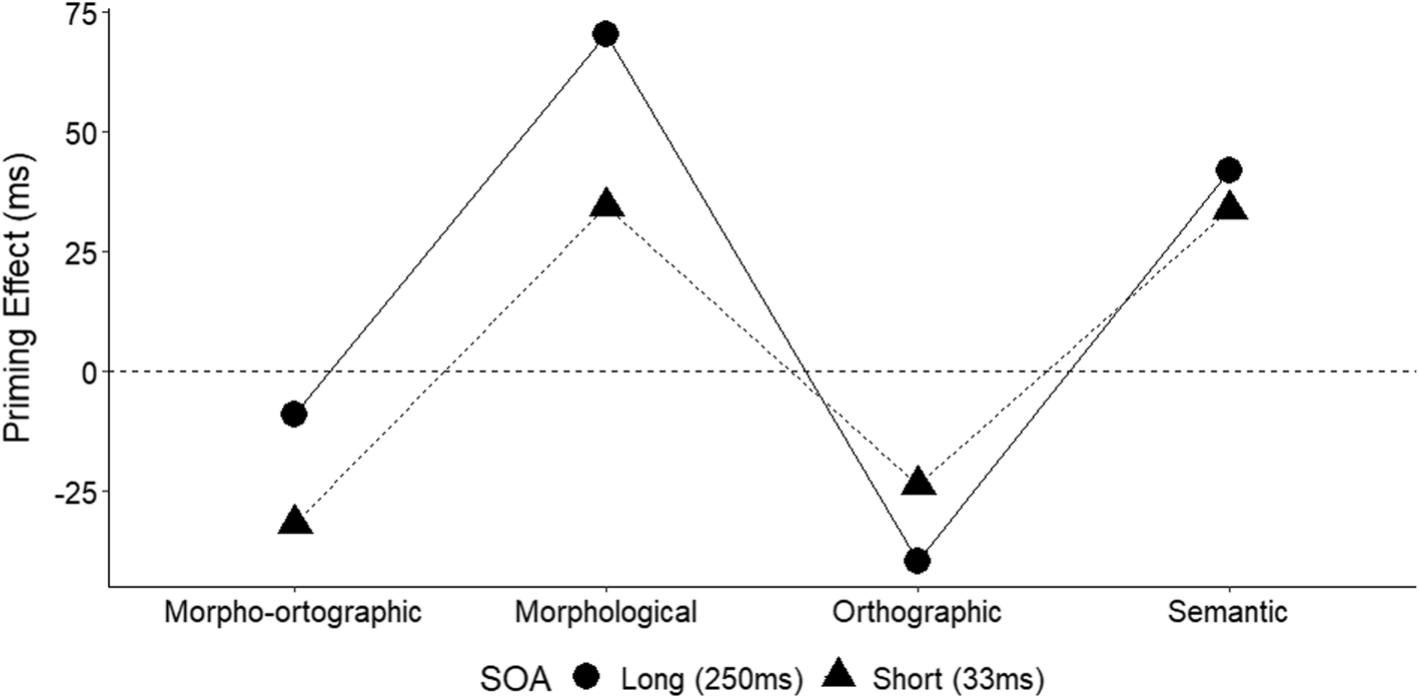
Priming effects by type of prime and SOA

When the SOA was 250 ms, facilitatory effects were observed for the morphological condition in comparison with the unrelated condition, Δ = 70 ms, *t*(39) = 6.281, *p* < 0.001, *d* = 0.993. The same pattern was observed between the semantic and the unrelated condition, Δ = 42 ms, *t*(39) = 3.848, *p* < 0.001, *d* = 0.608. In addition, a significant difference appears between morphological and semantic conditions, Δ = 29 ms, *t*(39) = 3.582, *p* < 0.001, *d* = 0.566, with items preceded by a prime with a morphological relationship being answered more quickly than items preceded by primes with only a semantic relationship. An inhibitory effect was identified for the orthographic condition in comparison with the unrelated condition, Δ = 39 ms, *t*(39) = 3.614, *p* < 0.001, *d* = 0.571, but no difference was found between the morpho-orthographic and the unrelated condition, Δ = 9 ms, *t*(39) = 0.614, *p* = 0.543, *d* = 0.097. Figure 1 shows the priming effects by prime type and SOA.

As the distribution of the proportion of error did not show normality in any of the experimental conditions by the Shapiro–Wilk test (all *p*s < 0.001), the non-parametric Friedman test was performed to assess the effect of the experimental conditions and the non-parametric Mann–Whitney test to assess the effect of SOA. Planned analyses were carried out using the non-parametric Wilcoxon test.

**Fig. 2:**
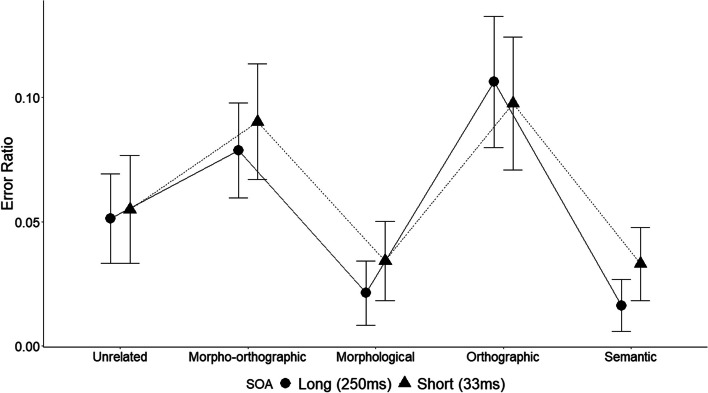
Error rates by prime type and SOA. Note. The error bars represent the 95% confidence interval

A statistically significant effect was identified for prime type, *χ*^2^(4) = 107, *p* < 0.001. However, not for SOA (*p* > 0.05). Planned analyses showed that when the SOA was 33 ms, no differences were found for the morphological and semantic condition in comparison with the unrelated condition (all *ps* > 0.08). Differences were found for the morpho-orthographic and orthographic conditions in comparison with the unrelated condition (all *ps* < 0.01), with the first two conditions showing more errors than the unrelated condition. When the SOA was 250 ms, a significant difference was observed between the morphological and semantic condition compared to the unrelated condition (all *ps* < 0.01). Again, a lower accuracy was observed for the morpho-orthographic and orthographic conditions compared to the unrelated condition (*ps* < 0.028). Figure 2 illustrates the difference in error rates among conditions.

Since we have identified significant facilitatory effects for both morphological and semantic conditions, we conducted correlation analysis to confirm semantic similarity’s effect on reaction times. Pearson correlations were performed between semantic similarity and reaction times logarithmically transformed. Medium and significant effects were observed for both SOA durations, 33 ms: *r* =  − 0.326, CI95% (− 0.491 to − 0.138), *p* < 0.001; 250 ms: *r* =  − 0.416, CI95% (− 0.556 to − 0.239), *p* < 0.001, indicating that words preceded by primes with high semantic similarity were answered faster.

## Discussion

The results demonstrated facilitation in visual recognition for words preceded by primes with a morphological or semantic relationship in the early processing stages. This facilitation was significantly higher than that observed for pairs of words that only shared an orthographic or morphological structure without a semantic relationship.

### Morphological and morpho-orthographic priming in Brazilian Portuguese

To determine whether the priming effect observed in the morphological condition is not due to the orthographic similarity between the prime and target, comparing this result with that of a control orthographic condition is essential. In this context, if facilitation effects in visual recognition are observed in the morphological but not in the orthographic condition, we can conclude by exclusion that the priming effect in the morphological condition is not due to mere orthographic overlap between the stimuli. 

Therefore, the results found for the morphological and orthographic conditions are in agreement with those observed in the literature, which suggests the role of morphological structures in the early stages of visual word recognition being facilitatory and independent of the orthographic similarity between words (Amenta & Crepaldi, 2012; Garcia et al., 2012; Hasenäcker et al., 2016; Lázaro et al., 2021; Quémart & Casalis, 2015; Quémart et al., 2011; Rastle & Davis, 2008; Rastle et al., 2000, 2004). These facilitatory effects of morphological structures also extended to the later processing stages, replicating the results of Rastle et al. (2000) and Quémart et al. (2011).

As for the morpho-orthographic condition, contrary to our hypotheses, no facilitation effects were observed in the initial stages of recognition when compared to an unrelated condition. Instead, an inhibitory effect was observed for the morpho-orthographic condition (e.g., solteiro [SINGLE]–solto [LOOSE]). Additionally, the effect observed for the morpho-orthographic condition in the short SOA condition was equivalent to that observed for the orthographic condition. These results differ from studies that suggest a morpho-orthographic segmentation process in the early stages of visual word recognition (Beyersmann et al., 2012, 2016; Grainger & Beyersmann, 2020; Heyer & Kornishova, 2018; Kazanina, 2011; Lázaro et al., 2021; McCormick et al., 2008; Mousikou & Schroeder, 2019; Quémart et al., 2011; Rastle & Davis, 2008; Rastle et al., 2000, 2004).

One of the first possible explanations for this discrepancy between our results and those observed in the literature is the linguistic differences and the way stimuli are constructed. Most studies that address the morpho-orthographic phenomenon have been carried out in English, a language with low grapheme-phoneme transparency and considered morphologically poor. Furthermore, many studies have used stimuli that can be decomposed, preserving the orthographic form of the suffix and the base (e.g., teacher = teach + er). On the other hand, in Portuguese, most of the derived words by suffixation do not preserve the orthographic form from which they originate, with frequent alterations of the thematic vowel (e.g., carteiro [MAILMAN] = carta [MAIL] + eiro). Thus, even if suffix recognition and decomposition occur, the remaining form will usually be a bound base (e.g., cart-) and not the form from which the derived word originated (e.g., carta [MAIL]). This process occurs both in the stimuli of the morphological condition and in the stimuli of the morpho-orthographic condition (e.g., solteiro [SINGLE] = solt- + eiro; cordeiro [LAMB] = cord- + eiro).

In the study by Neto and Dias (2014) with Brazilian Portuguese speakers, words formed by bound stems were answered significantly faster than words formed by free stems in a priming task, with morphological primes displayed for 100 ms. According to the authors, these results would indicate that words formed by bound stems do not undergo the decomposition process that occurs with words formed by free stems, so words formed by bound stems would be processed and stored in their entire form. This explanation provides a new perspective for the results found by Pinto (2017), in which no differences were observed between morphological primes (e.g., mineiro [MINER]–carteiro [MAILMAN]), morpho-orthographic primes (e.g., mineiro [MINER]–solteiro [SINGLE]) and unrelated primes (e.g., mineiro [MINER]–caneta [PEN]). It is possible that the absence of priming effects for the first two conditions occurred because the stimuli were bound stems and did not undergo the morphological decomposition process. Thus, not allowing the suffix decomposition that theoretically facilitates the recognition of the target word.

Considering that, it is possible that while stimuli of the morphological condition have benefited from the sharing of semantic representations to enable a more efficient recognition, eliciting the priming effects observed, the stimuli of the morpho-orthographic condition have been processed in their entirety without undergoing the decomposition process that would allow the extraction of the base, and consequently, facilitate the target word recognition. With the activation of the entire form of the prime in the morpho-orthographic condition and the absence of semantic relation between the prime and target, the representations of both stimuli would compete during visual recognition, hindering the target recognition and explaining the presence of inhibitory effects.

Another similar explanation for these findings is that stimuli of the morpho-orthographic condition have undergone a process of lexicalization. For morphology, lexicalization occurs when complex structures are transformed into simple ones through phonological, morphological, or semantic operations, which establish an arbitrary relationship between sound and meaning, hindering the complex word recognition by their constituents (Villalva, 2008). For example, the words used in the morpho-orthographic condition (e.g., solteiro [SINGLE], cordeiro [LAMB], and padrão [STANDARD]) have lost their compositional characteristics since their meaning cannot be accessed through their constituents, unlike the items of the morphological condition (e.g., barbeiro [BARBER], chaveiro [KEYCHAIN] and pianista [PIANIST]) that can be understood through the meaning of their parts. Therefore, it is possible that items used in the morpho-orthographic condition, once lexicalized, are not recognized through their constituents. Therefore, such items would be accessed via their entire form, not undergoing the morphological decomposition necessary to activate the morpho-orthographic representations that would facilitate the target word recognition.

### Semantic effects during visual word recognition

A curious result was the effects observed for the semantic condition. Although few studies have used a semantic condition when evaluating morphological processing, they have reported priming effects only when SOAs were greater than 250 ms (Quémart et al., 2011; Rastle et al., 2000). However, the results observed in our experiment only partially converge with those observed in the literature since semantic facilitation effects were also observed in the short SOA condition, suggesting the activation of semantic representations earlier than what was observed in other studies. Our results are in accordance with the studies by Crepaldi et al., (2010a), Diependaele et al. (2009) and Feldman et al. (2009), as well as other authors who have suggested the role of semantic information during the earliest stages of visual word recognition (Amenta et al., 2015; Beyersmann & Grainger, 2018; Grainger et al. 2020; Jared et al., 2017; Schimidtke et al. 2017).

Despite the results found by Rastle et al. (2000) and Quémart et al. (2011), studies that specifically investigated the role of semantic relations in visual recognition reported priming effects for SOAs of 28 ms in semantic categorization tasks (Bueno & Frenck-Mestre, 2008), and for SOAs of 66 ms (Perea & Rosa, 2002) and 67 ms (Perea, 1997; Perea et al., 1997) in lexical decision tasks. One factor behind the variability in the results is the distinction between semantic and associative relationships. A semantic relationship is understood as the overlapping of meaning or the number of characteristics shared between two concepts (e.g., duck and chicken are semantically related, as they share common characteristics, such as having feathers, wings, and the ability to fly) (Sánchez-Casas et al., 2012). Associative relationships, in turn, reflect the use of a word instead of the meaning, being this relationship established through the frequency that a word is used jointly with another (e.g., hair and head often appear together but do not share descriptive characteristics between themselves).

The study by Sánchez-Casas et al. (2012) controlled for these factors in a lexical decision study with SOA of 57 ms. Priming effects of 27 ms were observed for words that had a semantic relationship and were strongly associated (e.g., *mesa* [TABLE]–*silla* [CHAIR]), with no effect observed for pairs purely semantic (e.g., *hurácon* [HURRICANE]–*tormenta* [STORM]) or semantic with a weak level of association (e.g., *ojo* [EYE]–*gafas* [GLASSES]). The results of Sánchez-Casas et al. support the proposal of Moss et al. (1995) of an associative boost effect, in which greater priming effects would be found for pairs of words that are both semantically and associatively related. Thus, the semantic priming effect in short SOAs may reflect the automatic activation of stored representations of prime-related words. Meanwhile, the effect observed in longer SOAs (250 ms) reflects the use of a controlled process in a way that participants would wait for semantically related words. This being said, we may have found priming effects for the semantic and morphological conditions in the short SOA condition due to a strong semantic and associative relation between prime and target. This finding leads us to an important observation regarding studies on morphological processing. Few studies employ a semantic condition as a control for the morphological condition (e.g., Fleischhauer et al., 2021; Garcia et al., 2012; Quémart et al., 2011; Rastle et al., 2000), focusing their analyses only on the morphological, morpho-orthographic, and orthographic conditions. In addition, those studies that employ a morphological condition do not make explicit the level of association between prime and target words. Thus, the semantic relations and the association level between prime and target may be mediating the effects we have observed for the morphological pairs.

The picture drawn above may help in understanding the observed facilitative effects of the present study. In the morphological condition, derived words share orthographic, morphological, and semantic characteristics with the target word, which justifies using the semantic and orthographic conditions to isolate their respective effects, allowing for a more accurate evaluation of the impact of the morphological relationship in addition to orthographic and semantic similarities. Our results demonstrated that the facilitation observed for the morphological condition is not linked to a superficial orthographic similarity with the target word, since no facilitatory effect was observed for the orthographic condition compared to the unrelated condition. However, a priming effect was observed for the semantic condition. This fact hinders our interpretation of the results of the morphological condition, given that the items (prime and target) of this condition necessarily share semantic information. Thus, considering that morphological and semantic effects are additives, so that it would be possible to attest a morphological effect beyond the semantic one in the present study, we should have observed shorter reaction times and higher accuracy for the morphological compared to the semantic condition; however, this did not happen.

An alternative used in the literature to investigate the use of morphology during visual recognition is the employment of pseudowords consisting of morphemes in lexical decision tasks (Beyersmann et al., 2020; Burani et al., 2002; Caramazza et al., 1988; Crepaldi et al., 2010a; Dawson et al., 2018). This choice has the advantage of allowing greater control of variables underlying the stimuli and their constituents and allowing the isolation of the effect of semantic characteristics. Using pseudowords would allow investigating whether the activation of morphological structures occurs independently of lexical representation and, considering that pseudowords have no representation in the mental lexicon, would be forced to be processed through a decompositional route, as proposed in mixed models of processing, such as the Augmented Addressed Morphology Model proposed by Caramazza et al. (1988). Studies that employed this type of stimuli have found more convergent results regarding the role of morphology in visual word recognition by indicating longer reaction times and lower accuracy when pseudowords are composed of morphemes compared to items not constituted by morphological structures (Beyersmann et al., 2020; Burani et al., 2002; Caramazza et al., 1988; Crepaldi et al., 2010a; Dawson et al., 2018). Although studies using pseudowords in lexical decision tasks provide evidence of the use of morphology in visual word recognition, they do not replace priming studies, which are primarily interested in evaluating whether characteristics of prime can be decoded fast enough to interfere with target word recognition. In addition, the response to a stimulus in the lexical decision task, be it a word or a pseudoword, depends on the degree to which it differs from the stimuli of another class and not necessarily on its intrinsic characteristics (for an example, see the lexical decision tasks models proposed by Dufau et al., 2012; Norris, 2006; Ratcliff et al., 2004).

Finally, we observed that, regardless of the task used, there is a gap in studies in Brazilian Portuguese investigating the role of morphological structures in visual word recognition. Recently, Blasi et al. (2022) highlighted that most studies in cognitive science are conducted in English, which could contribute to bias in the area and an undue generalization of findings of English speakers to speakers of other languages. Our results diverge from the majority of the studies carried out so far about morpho-orthographic priming, not finding evidence of a morpho-orthographic segmentation process blind to semantic information in Brazilian Portuguese speakers. We observed instead the possibility that semantic representation can act in the early stages of visual word recognition, mediating morphological priming effects.

## Limitations and future studies

One limitation of our study was that it was not possible to control the length and frequency of stimuli used as primes completely. However, we believe these differences were insufficient to bias the results, given that neither variable showed significant correlations with reaction times in the short SOA condition.

Due to the low number of studies investigating the role of morphological structures in Brazilian Portuguese, we recommend that future studies seek to replicate the work carried out here, as well as alternatives for the analysis of morphological processing, such as the use of pseudowords as primes of target words or the use of low-frequency words, since these would tend to be processed through a decompositional route. We also point out the importance of including a semantic condition in the studies that evaluate morphological priming to verify whether the observed effects of the morphological condition are due to the presence of morphological structures or due to a semantic/associative relation between the prime and the target. Still, we raise the need for cross-linguistics studies, which enable the comparison of results obtained in Brazilian Portuguese with languages such as English and French, where the phenomenon has been frequently reported, using stimuli and tasks that are equivalent between languages. Finally, we recommend that future studies explore other morphological structures, such as prefixes and compounds, since these have different characteristics from words derived by suffixation, among them fully maintaining the orthographic form of the base.

## Conclusion

In recent years, the role of morphological structures during visual word recognition has been discussed, with studies suggesting a facilitation process stemming from the presence of morphological-related words. Additionally, a segmentation mechanism has been proposed that operates in the initial stages of visual recognition based solely on the appearance of morphological complexity of the stimuli and blind to the semantic and lexical information. In our study, no data supported this proposal of a morpho-orthographic segmentation phenomenon blind to semantic information. On the other hand, the semantic and associative relations present in the morphological items seem to mediate the priming effects. Therefore, it is not possible to determine to what degree morphological structures would be used in the early stages of visual word recognition. However, it should be noted that the results found here need to be replicated using different methodologies and morphological structures. We hope this work contributes to understanding how complex words and morphological structures are stored and processed in the mental lexicon in Brazilian Portuguese.

## Data Availability

The datasets generated and/or analyzed during the current study are available in the Open Science Framework (OSF) repository, https://osf.io/chp78/?view_only=9de1ac9423d74f36a341ca75db79665b

## Abbreviations

SOA: Stimulus Onset Asynchrony
ms: Milliseconds
SD: Standard deviation
DSCF: Dwass-Steel-Critchlow-Flinger
ANOVA: Analysis of variance
RT: Reaction time
CI: Confidence interval
